# A data set of earthquake bulletin and seismic waveforms for Ghana obtained by deep learning

**DOI:** 10.1016/j.dib.2023.108969

**Published:** 2023-02-11

**Authors:** Hamzeh Mohammadigheymasi, Nasrin Tavakolizadeh, Luís Matias, S. Mostafa Mousavi, Yahya Moradichaloshtori, Seyed Jalaleddin Mousavirad, Rui Fernandes

**Affiliations:** a Instituto Dom Luiz (IDL), Universidade da Beira Interior, Covilha, 6201-001, Portugal; b Departamento de Informatica, Universidade da Beira Interior, Covilha, 6201-001, Portugal; c Department of Geophysics, Stanford University, Stanford, CA 94305-2215, United States; d Instituto Dom Luiz, Faculdade de Ciencias, Universidade de Lisboa, Lisboa, 1749-016, Portugal; e Institute of Geophysics, University of Tehran, Tehran, 14359-44411, Iran

**Keywords:** Earthquake waveforms, Deep learning, Seismic catalog, Live Matlab figures

## Abstract

The Ghana Digital Seismic Network (GHDSN) data, with six broadband sensors, operating in southern Ghana for two years (2012-2014). The recorded dataset is processed for simultaneous event detection and phase picking by a Deep Learning (DL) model, the EQTransformer tool. Here, the detected earthquakes consisting of supporting data, waveforms (including P and S arrival phases), and earthquake bulletin are presented. The bulletin includes the 559 arrival times (292 P and 267 S phases) and waveforms of the 73 local earthquakes in SEISAN format. The supporting data encompasses the preliminary crustal velocity models obtained from the joint inversion analysis of the detected hypocentral parameters. These parameters comprised of a 6- layer model of the crustal velocity (Vp and Vp/Vs ratio), incident time sequence, and statistical analysis of the detected earthquakes and hypocentral parameters analyzed and relocated by the updated crustal velocity and graphic representation of them a 3D live figure enlighting the seismogenic depth of the region. This dataset has a unique appeal for earth science specialists to analyze and reprocess the detected waveforms and characterize the seismogenic sources and active faults in Ghana. The metadata and waveforms have been deposited at the Mendeley Data repository [1].

**Specification Table**

**Table:** 

Subject	Earth and Planetary Science; Geophysics
Specific subject area	Geophysics, seismology
Type of data	Formats: (1) miniseed (waveforms) (1) SAC (response files) (3) excel file (Updated Catalog) (4) Matlab figure (.fig) (5) ASCII format (Bulletin SEISAN format)
How data were acquired	The current data set is a set of earthquake waveforms that have been detected and extracted from the [3] dataset by applying the DL method and a “conservative strategy”. In addition to the extracted earthquake waveforms, an updated earthquake catalog up to July 2022 is provided for the Ghana region. The data also contains a 3D map and live figures with the (.fig) Matlab format for the readers to provide a 3D presentation of active seismogenic sources in Southern Ghana.
Data format	Analyzed and filtered
Data source location	Country: Ghana Primary data source: Ghana Digital Seismic Network Latitude: [5°0′00”, 7°0′00”], Longitude: [1°30′00”, 0°30′00”]
Data accessibility	The processed dataset has been deposited in the Mendeley repository and is accessible using the link: https://data.mendeley.com/datasets/zz9txhw89w/1 DOI:10.17632/zz9txhw89w.1[1]
Related research article	H. Mohammadigheymasi, N. Tavakolizadeh, L. Matias, S.M. Mousavi, G. Silveira, S. Custodio, N. Dias, R. Fernandes, Y. Moradichaloshtori, Geosystems and Geoenvironment 2(2), 100152 (2023) [2].

## Value of the Data

• The seismic waveforms, the crustal velocity data, and the updated catalog inspire Earth Science researchers to conduct more advanced studies on the seismicity and tectonics of the region.
• The seismic catalog data updated to July 2022 can be used as a source to conduct Probabilistic Seismic Hazard Assessment (PSHA) on the region. This is specifically important because of the lack of a complete source of seismic data in the region.
• The 3D live figures of the seismicity in Southern Ghana provide a 3D insight into the active seismogenic sources in Southern Ghana.

## 1 Objective

This article provides a detailed explanation and supplementary information on an original paper [2], including earthquake waveforms, the compiled seismic catalog, the quality parameter of events, the seismicity parameter estimates, the 3D hypocentral map, and some preliminary analyzes of the seismicity analysis performed. The earthquake waveforms are provided in Seisan format as an easy source for additional studies, such as focal mechanism studies. The performed catalog is a ready resource for conducting seismic hazard assessments. This paper enhances the original paper by providing additional analysis and detailed information.

## 2 Data Description

Massive digital seismic data were recorded by GHDSN by broadband sensors equipped with GPS-clock timing between September 2012 and April 2014. Local earthquakes in this dataset were detected using DL methods [3]. The compiled earthquake catalogs from the processing GHDSN dataset [4] include 73 events. Post-processing was performed on the detected earthquakes to obtain an updated crustal velocity model and hypocentral parameters of the detected earthquakes [5,6]. Here, we present detailed information about the detected earthquakes, their supplementary material, and live figures graphically depicting the active seismogenic sources in this region. (Files and folders shared at Mendeley Data repository: Hypocenters_fig6.fig file; Ghana-catalog_update-2022-04.csv file; Data-Availability-Table folder; Data folder; README.md file) [1].

### 2.1 Compiled Updated Catalog

The compiled catalog file from the dataset, (presented in CSV format) contains 11 columns; each row contains information about an individual earthquake, while the columns represent the associated parameters. The catalog file is presented in the standard input format for common Probabilistic Seismic Hazard Assessment (PSHA) software such as OpenQuake [7]. The catalog contains the following attributes:

1. 1: event ID; 2: YEAR; 3: MONTH; 4: DAY: ID and date type variables indicating the date for each earthquake.
2. 5: HOUR; 6: MINUTE; 7: SECOND: date type variables indicating the time for each earthquake.
3. 8: LATITUDE; 9: LONGITUDE: double type variables (three decimal digits) indicating the location (longitude and latitude) for each event.
4. 10: DEPTH: double type variable (one decimal digit) indicating the depth of each event.
5. 11: MAGNITUDE: double type variables indicating the reported magnitudes (one decimal digit) for the included earthquakes. The type of the magnitude is Ml (Local magnitudes) for the newly found events and Mw (Moment Magnitude) for other compiled events.

**Fig. 1:**
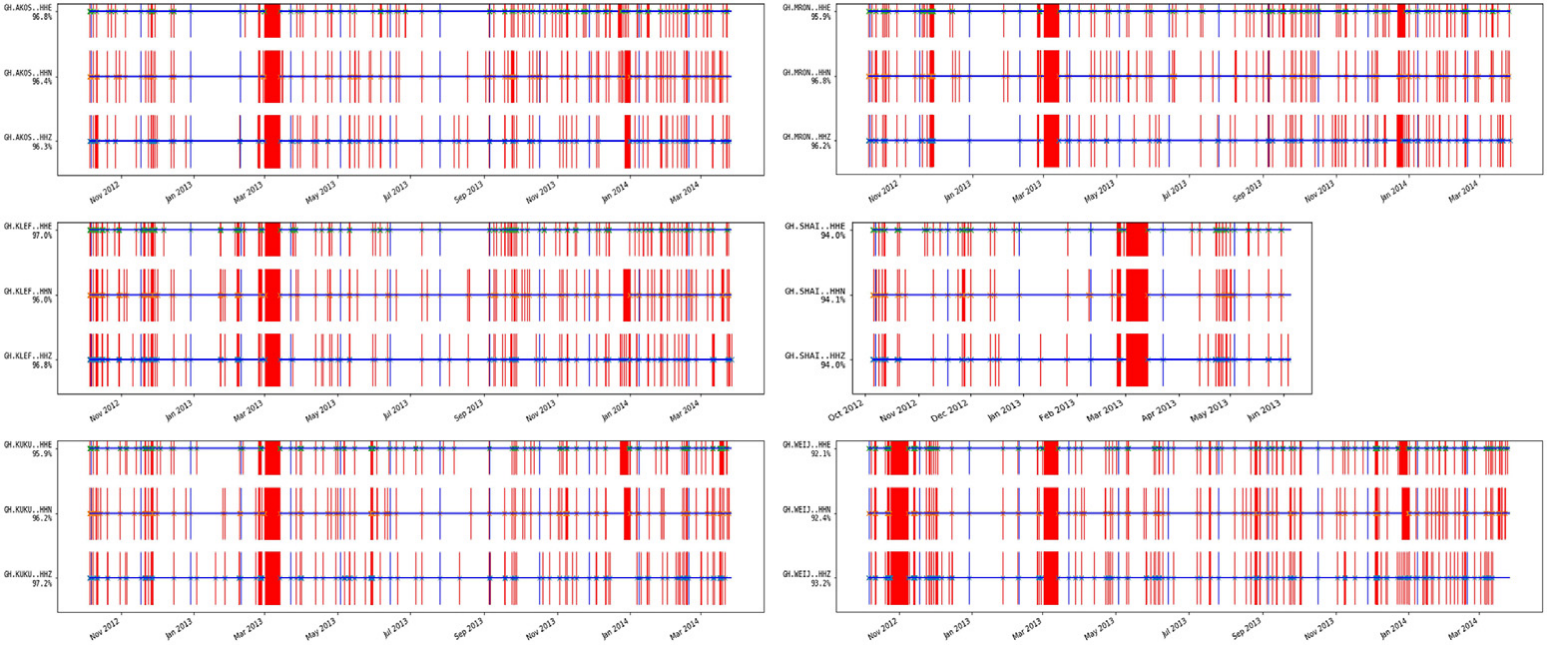
Data availability timetable for the stations in the Ghana Digital Seismic Network.

**Fig. 2:**
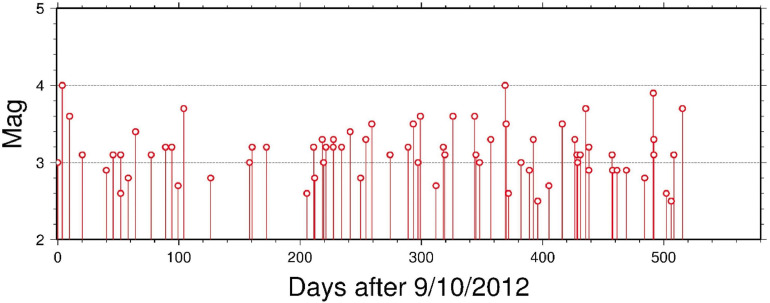
The figure shows the time sequence of detected events by the DL method. As can be seen, there is no cluster of events in time.

**Fig. 3:**
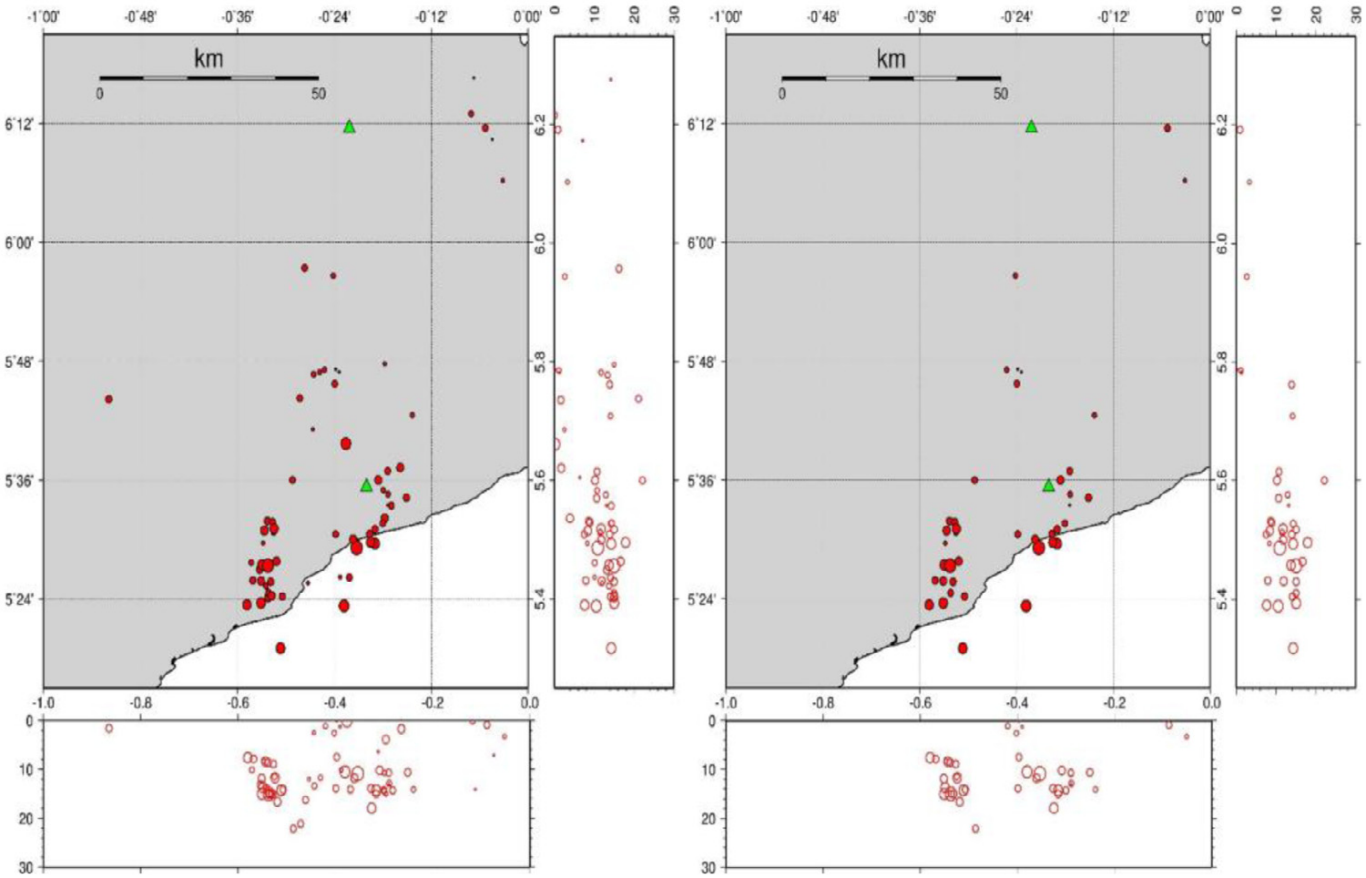
Map of the set of events: left panel) the complete set of events, right panel) a subset of the better-quality events with RMS ≤ 0.5 and NS TA ≥ 4, containing 43 events.

**Fig. 4:**
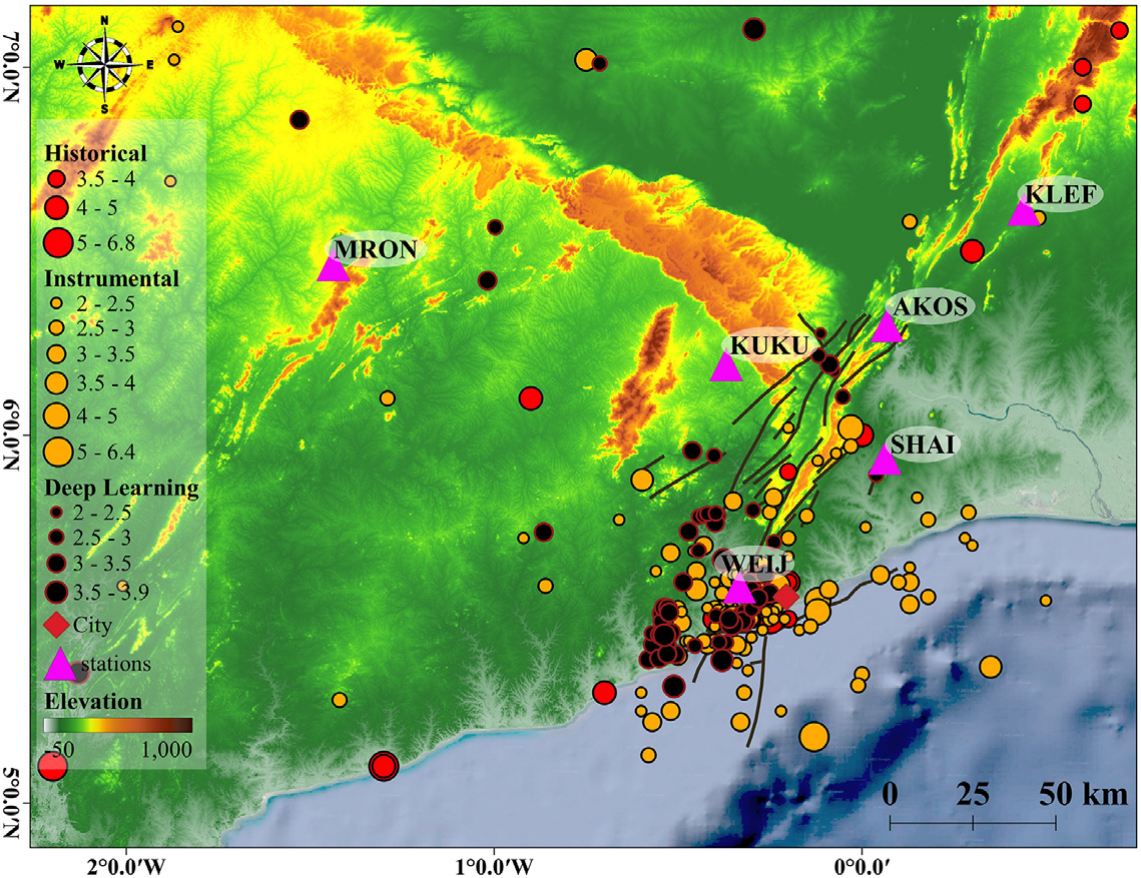
The epicentral map of updated catalog for Ghana and the surrounding area, including the historical, reported instrumental, and the DL catalogs. In addition to these clustered events, there is scattered seismicity that correlates with the AFZ. The depth distribution of the event ranges [0, 25] km with a focus on the [5, 15] km. As a result of locating events by the updated velocity model, the events are highly concentrated and correlated with the mapped fault zones and the depth distribution is confined to the upper crust.

**Fig. 5:**
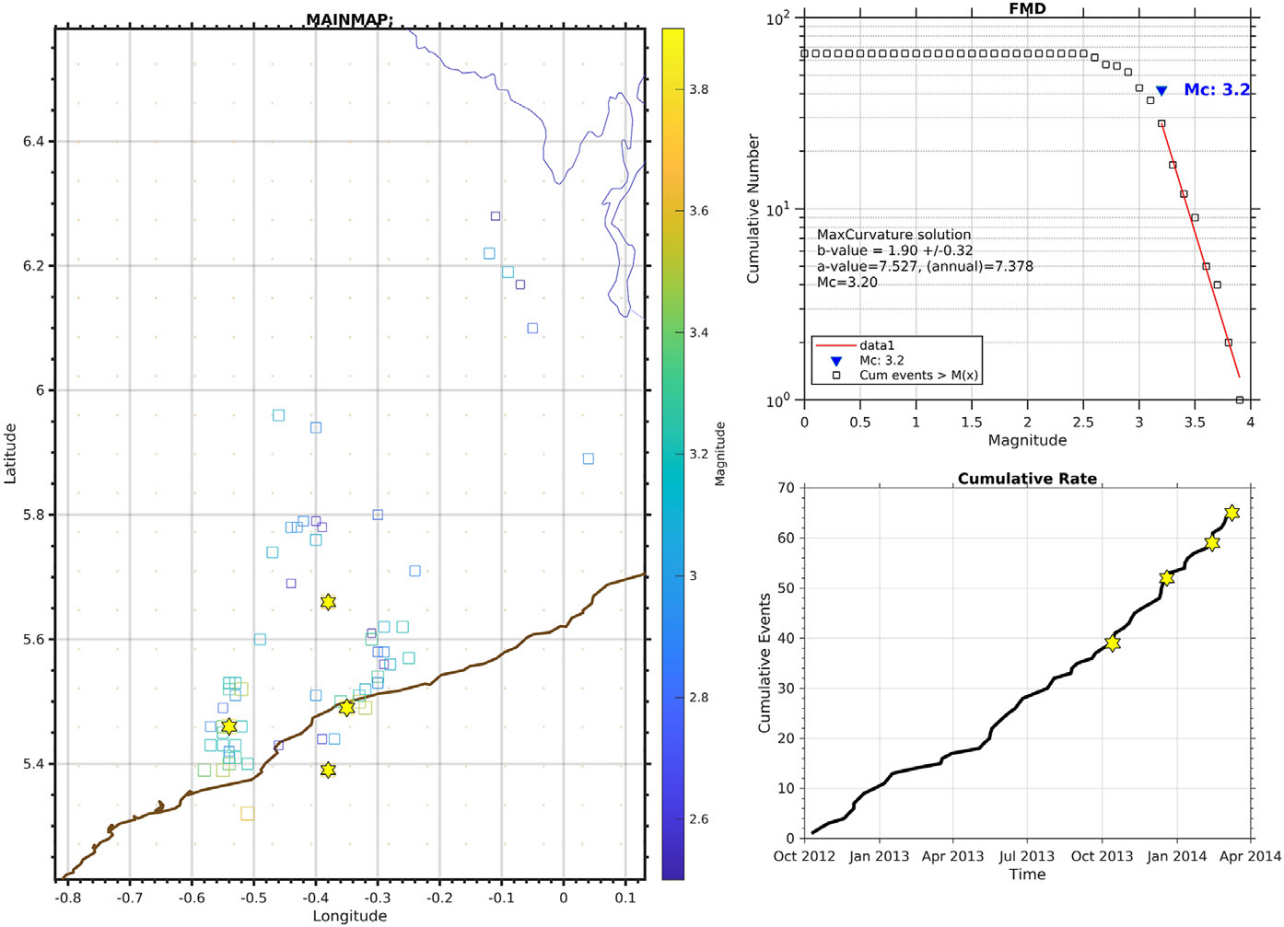
Left: Epicentral map, Top right: Cumulative curve as a function of magnitude, Bottom right: Cumulative number of events as a function of time. The figures are generated by using ZMAP software [9].

The data availability timetable of the raw GHDSN dataset is shown in Fig. (1). A time sequence plot of the detected earthquakes in this dataset is shown in Fig. (2). The detected events are classified based on different criteria presented in Table 1, Table 2, Table 3. Two sets of events classified as the complete set and a subset with a higher detection accuracy are shown in Fig. (3) [see Sec. (3) for more details]. A total number of 303 earthquakes, compiled from data centers, published articles, local institutes, and the detected events in [2] are depicted in Fig. (4). Finally, a magnitude of completeness and b-value plot of the detected events is shown in Fig. (5).

**Table 1:** Number of events with GAP less than or equal to.

GAP	120	140	160	180	200	220	240	260	280	300	320	340
N	0	4	9	12	14	21	26	39	62	65	68	71

**Table 2:** Number of events with RMS less than or equal to.

RMS	0.1	0.2	0.3	0.4	0.5	0.6	0.8	1.0	1.6	2.0	3.0	4.0	5.0	13.0
N	19	37	49	56	60	61	63	66	67	68	70	71	72	73

**Table 3:** Number of events with a number of stations equal or greater than.

NSTA	2	3	4	5	6	7
N	73	72	55	30	6	0

### 2.2 Seismic Waveforms and Bulletin in the Seisan Format

A set of earthquake waveforms for 73 detected earthquakes, accompanied by its earthquake Bulletin information, is provided in the Seisan format (S-file) [8].

### 2.3 Live 3D Matlab Figures of Hypocenters

This live 3D figure in MATLAB format (“Hypocenters_fig6.fig” file in [1]) shows the hypocentral positions calculated using the joint-inversion method. Different earthquake clusters are shown in distinct colors, permitting recognizable delineation of the seismic sources. The live feature of the images lets the operator rotate and evaluate the hypocenter locations from different angles.

### 2.4 Updated Crustal Velocity Model in SEISAN Format

The updated crustal velocity model in SEISAN format consists of 6 constant velocity layers. The top layers are 1, 13, 8, 13, and 10km thick, corresponding to V_p_ = 5.9, 6.1, 6.3, 6.5, and 6.9km/s, respectively. A halfspace layer with a velocity of 7.2km/s underlies the model.

## 3 Experimental Design, Materials and Methods

The current data set is a set of earthquake waveforms, seismic catalog, and detailed earthquake Bulletin that has been detected and extracted from the [4] data set by applying the DL method and a ”conservative strategy”. In addition to the extracted earthquake waveforms, an updated earthquake catalog up to July 2022 is provided for the Ghana region.

The data also contains a 3D map and live figures with the (.fig) Matlab format for the readers to provide a 3D presentation of active seismogenic sources in Southern Ghana. The network recorded earthquake waveforms of local and Teleseismic earthquakes in this time period. The detected earthquake in this time interval amenable to locate is shown in Fig. (4). This figure also shows the compiled catalog of all the data sources (referred to in Sec. (2)).

The related article entitled “Application of deep learning for seismicity analysis in Ghana” has been published in the Geosystems and Geoenvironment journal.

### 3.1 Characterization of Detected Events and Quality Parameters

#### 3.1.1 Time Sequence of the Earthquakes

The hypocentral parameters of the earthquakes are estimated by applying a joint-inversion algorithm to search simultaneously for the ideal velocity model and the hypocentral parameters that best fit the arrival times [8]. Here, we briefly present the characterization of these events. Fig. (2) shows a homogeneously time sequence of the earthquakes over time, implying that there is no time cluster of events during this period.

### 3.2 Other Quality Parameters

As another quality control parameter to evaluate the accuracy of the detected events, here, we present information about

1. Number of events in the specified GAPs
2. Number of events with specified Root Mean Square Error (RMSE)
3. Number of events with a number of stations equal to or greater than, in Tables 1, 2, and 3, respectively.

### 3.3 Depth Distribution and Clusters

According to the quality statistics presented in the previous section, we classified the events into two sets: 1) the complete set, and 2) a subset of the better-quality events with RMS ≤ 0.5 and NS TA ≥ 4, which contains 43 events.

### 3.4 Plot of Full Catalog Updated to April 2022

The epicentral map of the updated catalog for Ghana and the surrounding area, including the historical, reported instrumental, and the DL catalogs, are plotted in Fig. (4).

### 3.5 Magnitude of Completeness and b-Value Calculation

In this part, we present the magnitude of completeness and b-value of the catalog containing 73 detected earthquakes by DL method. The MC for 73 detected events by DL is shown in Fig. (5). Accordingly, the Frequency Magnitude Distribution (FMD) and the cumulative rate show the standard pattern with Mc=3.2 and b-value=1.9.

## Ethics Statements

Not applicable.

## CRediT Author Statement

**Hamzeh Mohammadigheymasi:** Methodology, Software, Data curation, Writing – original draft, Visualization, Conceptualization, Interpretation; **Nasrin Tavakolizadeh:** Methodology, Software, Data curation, Writing – original draft, Visualization, Interpretation; **Luís Matias:** Methodology, Software, Supervision; **Yahya Moradichaloshtori:** Writing – original draft, Methodology, Software; **S. Mostafa Mousavi:** Methodology, Software; **Seyed Jalaleddin Mousavirad:** Software; **Rui Fernandes:** Supervision, Funding acquisition.

## Declaration of Competing Interest

The authors declare that they have no known competing financial interests or personal relationships that could have appeared to influence the work reported in this paper.

## Data Availability

Seismicity dataset of Ghana obtained by Deep Learning (Reference data) (Mendeley Data).

## Appendix Supplementary materials

Image, application 1
